# A Cadaveric Study on the High Origin of the Radial Artery

**DOI:** 10.7759/cureus.52595

**Published:** 2024-01-19

**Authors:** Shilpa S, Rani Raphael M, Rajad R, Manju S, Tejaswi H Lokanathan

**Affiliations:** 1 Anatomy, Government T D Medical College, Alappuzha, IND; 2 Anatomy, Government Medical College, Kollam, IND; 3 Anatomy, Adichunchanagiri Institute of Medical Sciences, B.G Nagara, IND

**Keywords:** clinical relevance, cadaver, upper extremity, cross-sectional studies, axillary artery, radial artery

## Abstract

Introduction: Among the upper limb's vascular variations, the radial artery's high origin from the axillary artery is rare, and literature regarding the same is limited. Anomalous origin of radial artery can cause failure of radial approach to coronary angiography and reconstructive surgeries of upper limbs and hence is of clinical significance. With this background, the current cadaveric study was planned to describe the branching pattern of the axillary artery and its variations.

Methods: We conducted this descriptive, cross-sectional study on sixty adult human cadaveric upper limbs at the anatomy departments of Government TD Medical College, Alappuzha, and Government Medical College, Thiruvananthapuram, over two years from 2021 to 2023. The axillary artery's branching pattern and termination were noted, and the prevalence of high origin of the radial artery from the axillary artery was documented.

Results: High origin of radial artery from axillary artery was observed in four (6.70%) limbs and was higher than the prevalence reported in earlier literature. Among these variations, one was a female cadaver with a bilateral high origin of radial artery arising from the third part of the axillary artery. The other two were from separate male upper limbs, both from the right upper limb.

Conclusion: The prevalence of the high origin of the radial artery from the axillary artery was high compared to earlier reported literature. This calls for further research in the anatomy of arterial patterns of the upper limb to avoid complications during arterial procedures of the upper limb.

## Introduction

The axial artery of the upper limb is the subclavian artery (SCA), which continues as the axillary artery (AA) from the outer border of the first rib to the lower border of the teres major and after that courses as the brachial artery (BA). The BA divides into its terminal branches, the radial artery (RA) and the ulnar artery (UA), in the cubital fossa at the level of the neck of the radius [1-5]. 

The pectoralis minor (PM) muscle divides the AA into three parts. From the first part of the AA proximal to PM arises the only branch, the superior thoracic artery (STA); from the second part of the AA behind PM arises the thoracoacromial artery (TA) and the lateral thoracic artery (LTA) and from the third part of the AA distal to the PM arises the subscapular artery (SSA), anterior circumflex humeral artery (ACHA) and posterior circumflex humeral artery (PCHA) [1-5].

Anatomic variations in AA branching patterns are not uncommon and typically involve the SSA and PCHA. Sometimes, anomalous high division of the AA divides into RA and UA and is occasionally also the source of the anterior interosseous artery (AIA) [1].

The vascular variations of upper limbs are always a concern for anatomists, surgeons, and interventional radiologists. Anatomical variations in the vascular pattern of the upper limb are encountered during routine cadaveric dissections as well as surgical and radiological interventions. The literature describes 11-24.4% of individuals as having variations in major upper limb arteries [6].

Knowledge about these foreseeable variations in arterial anatomy of the upper limb is essential to avoid accidental iatrogenic injuries during diagnostic interventions and surgical treatments involving the upper limb. With this background, the current study was planned to observe and document the branching pattern of the AA and the high origin of RA from the AA in human cadavers.

## Materials and methods

The institutional ethics committee of Government TD Medical College, Vandanam, Alappuzha, approved the study [letter number EC:10/2021 dated 29/03/2021]. Sixty formalin-fixed cadaveric upper limbs were studied in this cross-sectional study. These thirty upper limbs from the right and left side each were collected from 24 adult male and six adult female cadavers. All cadavers included in the research were donated to the anatomy departments of the Government T D Medical College, Vandanam, Alappuzha, and the Government Medical College, Thiruvananthapuram, Kerala, through a voluntary body donation program.

Inclusion Criteria

Adult human cadavers in the age group 60 years to 80 years, with documented non-medicolegal causes of death and received through the voluntary body donation program, were included in the study. 

Exclusion Criteria

Adult human cadavers in the age group of 60 years to 80 years but with previous history suggestive of vascular anomalies, scars over the upper limb region, signs of decomposition, and pathologic lesions over the upper limbs were excluded from the study. Cadavers with unknown causes of death and also medicolegal causes were excluded from the study. 

The dissection of the AA was carried out according to methods described in Cunningham's Manual of Practical Anatomy Volume 1, 16th edition [7]. Four incisions were made to reflect the skin over the pectoral region and axilla. The first was a vertical incision from the suprasternal notch to the xiphisternum. The second incision was from the suprasternal notch to the acromial end of the clavicle on both sides along the course of the clavicle. The third incision was an oblique incision from the xiphisternum to the nipple, encircling the nipple and areola, and further extended upwards to the lateral wall of the axilla at the level of insertion of teres major. The last was a horizontal incision from the xiphisternum to the midaxillary line. Following incisions, the skin flaps were reflected laterally to expose the pectoralis major.

Further, the pectoralis major was incised from its clavicular and sternal attachment and reflected towards its insertion to the lateral lip of the bicipital groove of the humerus. The area medial to the upper border of pectoralis minor and lateral to the lower border of pectoralis major was cleaned to expose the origin and termination of the axillary artery. Pectoralis minor was detached from its origin and reflected towards its insertion into the coracoid process. The branching pattern of the axillary artery was observed, and the number of direct branches from the AA was noted. Observations regarding the origin of various branches were documented and photographed. The artery was highlighted as an when required.

In the forearm, the brachioradialis muscle was reflected laterally to observe the RA, and the artery's origin was noted. In cases of a high origin, the RA was traced to identify the source. In the hand, the palmar aponeurosis was cut and reflected to trace the course of the superficial palmar branch of the RA. The artery was then traced dorsally, and branches such as the dorsal carpal branch and the 1st dorsal metacarpal artery were studied. The course of the artery through the 1st dorsal interossei and its branches in the palm, including the princeps pollicis artery and radialis indicis artery, were observed. Finally, the adductor pollicis muscle was cut to examine the artery's final course and completion as the deep palmar arch.

Statistical Analysis

The prevalence of high origin of RA was reported as a percentage. 

## Results

Among the 60 upper limbs studied, four (6.70%) upper limbs showed a high origin of the RA from the AA. Among these variations, one was a female cadaver with a bilateral high origin of the RA from the third part of the AA. The other two limbs were cases of high origin of the RA from 2nd part of AA and were from two separate male cadavers. In all these cases, the RA originated above the junction of the median nerve's two roots (MN) roots and showed no cubital crossovers. All four limbs invariably showed a similar course and relation of the RA to the MN in the arm and cubital fossa and gave off all the branches as typical RA. In any of these cases, no unusual branches were observed in the arm from the brachioradial artery. The AA continued as the BA, which terminated by dividing into UA and common interosseous arteries in all four limbs. In all cases, the superficial palmar arch was completed by a well-developed superficial palmar branch of the radial (brachioradial) artery. The formation of the deep palmar arch was also observed to be normal.

**Figure 1:**
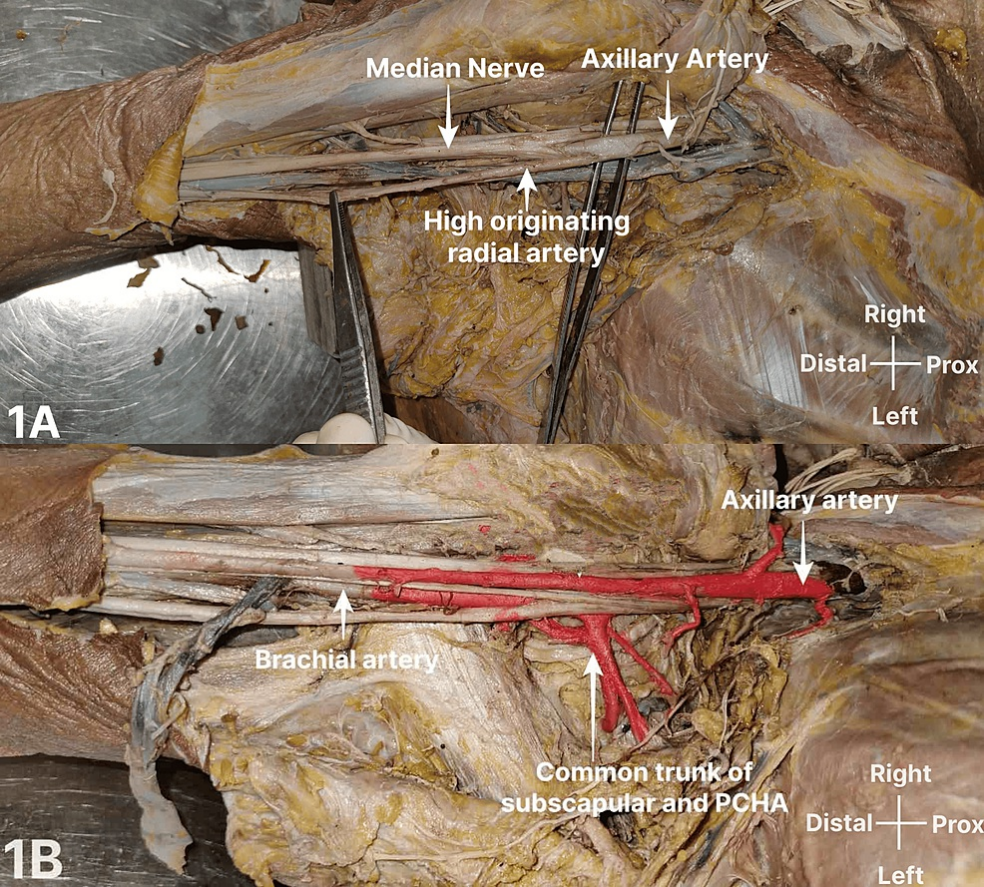
High origin of the RA from the third part of the AA This branching pattern was observed in the right upper limb of a female cadaver aged about 70 years, collected from Government TD Medical College, Vandanam, Alappuzha. Figures 1A-1B are from the same cadaver. Figure 1A shows a high-originating RA from the third part of the AA. Figure 1B shows the common trunk of the SCA and PCHA from the third part of the AA visualized after the removal of the axillary vein. RA: radial artery; AA: axillary artery; SCA: subclavian artery; PCHA: posterior circumflex humeral artery

The first case (Figure 1A, 1B) was that of a female cadaver, where we observed an unusual branch from the third part/infrapectoral part of the AA in both upper limbs. On the right side, the first and second parts of the AA were normal, whereas the third part was divided into deep and superficial branches. The deep branch was posterior to the MN and gave rise to a common trunk, from which arose the SSA, PCHA, and extra upper subscapular branch accompanying the nerve of the same name. The ACHA also emerged from the deep branch and continued downwards as the BA, which in turn gave off the profunda brachii and other branches of BA in the arm. The superficial branch arose before the junction of two roots of MN and was lying anteromedial to the MN as well as the axillary vein and had no branches in the arm, descended downwards to the arm along the medial aspect of MN, and crossed the MN from medial to lateral anteriorly in middle of the arm.

**Figure 2:**
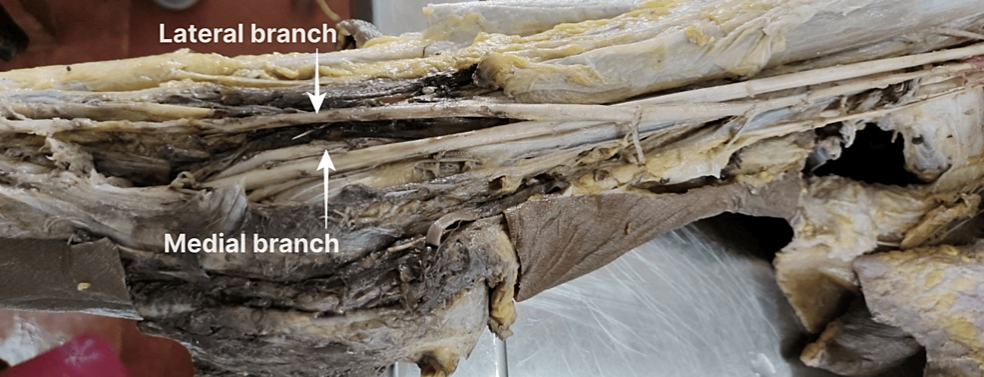
Course of high origin of the RA from the third part of the AA in the cubital fossa This branching pattern was observed in the first case, as shown in Figure 1, i.e., the right upper limb of a female cadaver aged about 70 years, collected from Government TD Medical College, Vandanam, Alappuzha. RA: radial artery; AA: axillary artery

In the cubital fossa, the lateral branch (Figure 2), following the normal course of the BA in the arm, ended by continuing as RA, deep to the deep head of pronator teres. The medial branch continued as the UA deep to the bicipital aponeurosis and along the lateral aspect of the forearm deep to the brachioradialis. Its course and branches were the same as that of a normal RA in the forearm and hand and terminated by anastomosing with the UA to form the superficial and deep palmar arches. 

**Figure 3:**
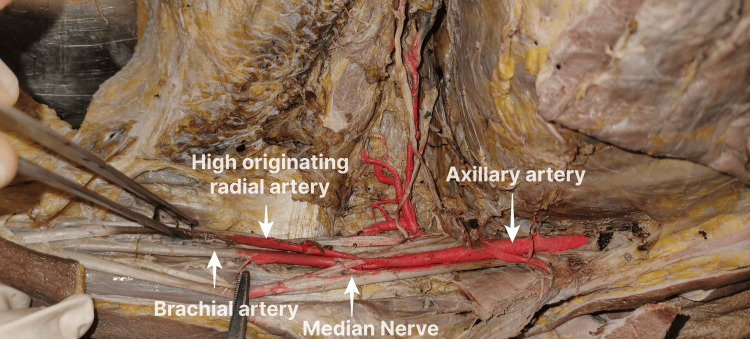
High origin of the RA from the AA This branching pattern was observed in the left upper limb of the same female cadaver (as in Figure 1), aged about 70 years, collected from the Government TD Medical College, Vandanam, Alappuzha. RA: radial artery; AA: axillary artery

The left upper limb of the same cadaver (Figure 3) also showed a high origin of the RA from AA above the junction of two roots of the MN. Here, the first and second parts of the AA were normal, but the third part gave off a common trunk for SSA and PCHA. ACHA arose from the SSA's circumflex scapular branch in this limb. After the origin of the common trunk, the AA was divided into medial and lateral branches. Both branches had a similar course, relation, and branches as the other limb, but the medial branch, the RA with a high origin, was constricted at its origin. Its lumen was of a normal caliber in its course downwards.

**Figure 4:**
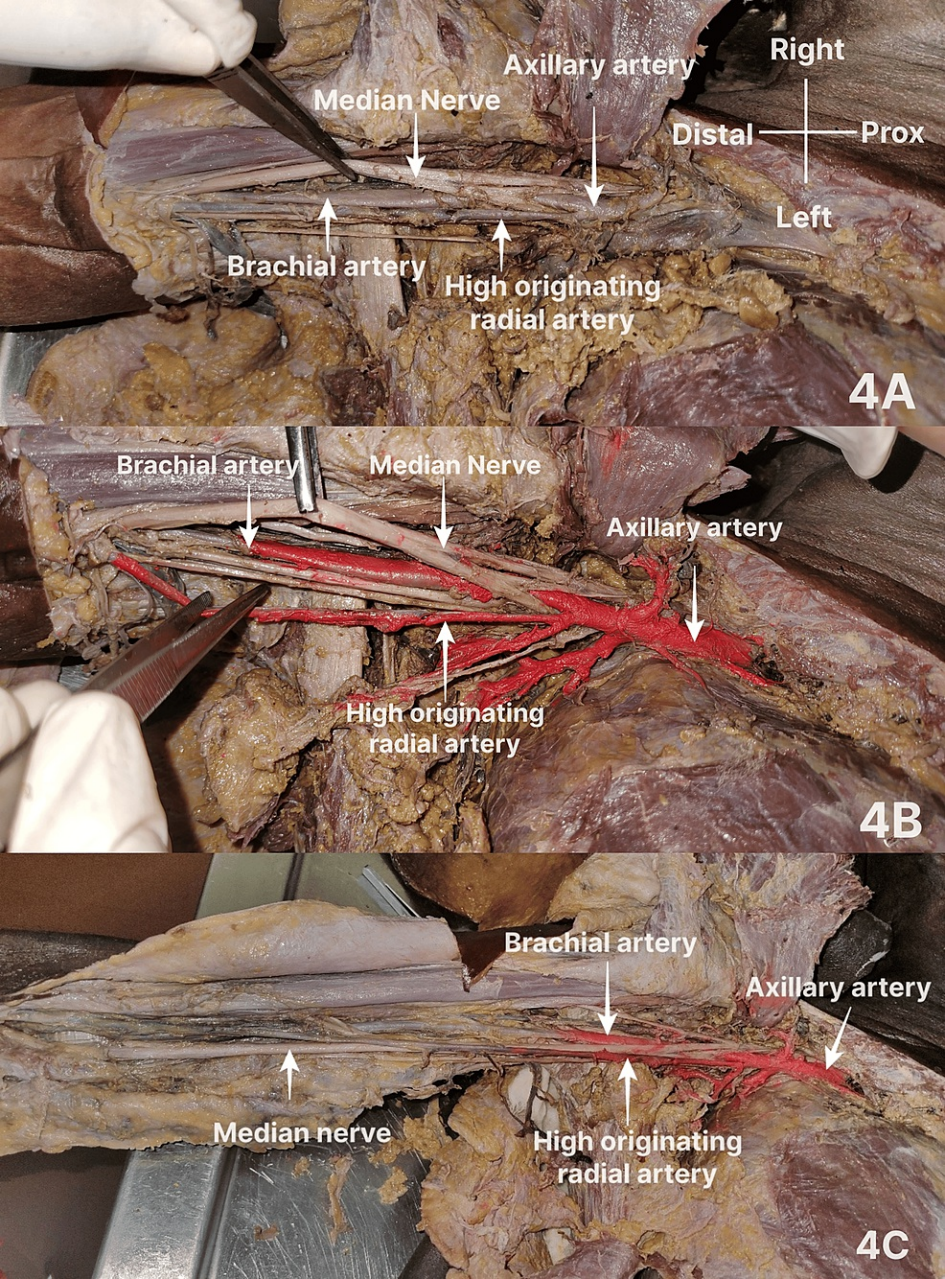
High origin of RA from the second part of the AA This branching pattern was observed in the right upper limb of a male cadaver aged 62 years, collected from Government T D Medical College, Vandanam, Alappuzha. Figures 4A-4C are from the same cadaver. Figure 3A shows the high-originating RA from the second part of the AA. Figure 3B shows other branches of the AA after further cleaning. Figure 3C demonstrates the in situ relation of the MN to the high-originating RA. RA: radial artery; AA: axillary artery

The next similar case (Figure 4) was observed in the right upper limb of a male cadaver. Here, the second part of the AA gave rise to TA, LTA, and SSA. After these branches, a common trunk arose, which gave rise to the thoracodorsal artery and the RA. The third part of the AA gave rise to ACHA and PCHA and continued into the arm as the BA, which in turn continued as UA. In this case, the common trunk arose before the junction of two roots of the MN. The common trunk from the second part of the AA, after giving off the thoracodorsal artery which accompanied its fellow nerve to supply the latissimus dorsi muscle, continued anteromedial to the MN and axillary vein to the arm, crossed the MN anteriorly to lie in its lateral aspect in the cubital fossa deep to the bicipital aponeurosis. No cubital crossover was observed. It continued deep to the brachioradialis to the wrist. It was divided into superficial and deep branches, which completed the respective arterial arches of the palm by anastomosing with terminal branches of UA.

**Figure 5:**
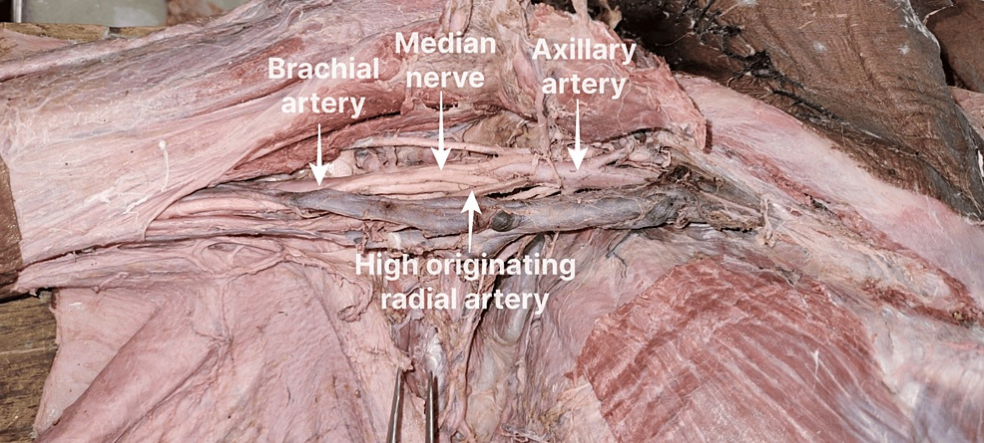
High origin RA from the second part of AA This branching pattern was observed in the right upper limb of a male cadaver aged 67 years, collected from Government Medical College, Thiruvananthapuram. RA: radial artery; AA: axillary artery

The fourth limb (Figure 5) with similar variation was on the right side of another male cadaver. The aberrant artery originated from the second part of the AA, coursed superficially into the arm, crossed the cubital fossa, continued along the lateral aspect of the forearm, and terminated in the palm by forming the deep palmar arch. The BA continued as the UA in the cubital fossa.

## Discussion

The present study showed a high origin of RA from the AA in 6.70% of the studied upper limbs. It accounted for 10% (three out of 30) of the studied cadavers: one female cadaver with bilateral variation and two male cadavers with variation on one side. Among the vascular anomalies of the upper limb, the high origin of the RA from the AA is rare, and literature regarding the same is limited. High-originating RA is referred to as brachioradial artery in literature. The brachioradial artery comprises all those radial arteries that arise high above the level of its normally described origin, i.e., one cm distal to the elbow joint at the level of the neck of the radius.

In a study on 750 upper limbs, McCormack reported that RA originated from the AA in 14 left and two right upper limbs [8]. Uglietta and Kadir [6] reviewed 100 upper extremity arteriograms and found the RA and the UA origin from the AA to be infrequent, accounting for only 2% of the observed arteriograms. 

In 1991, Compta [9] reported a case of bilateral high-origin RA from the AA associated with hand vascular anomalies. Compta mentioned that a high-originating RA includes those arising from the BA and the AA. Various authors often consider it a persistent superficial brachial artery (SBA) [10-13]. Compta described them to be two distinct anatomical elements. He explained that the persistent SBA emerges from the AA or the BA and divides into the RA and UA in the forearm, is generally superficial, and the BA supplies the interosseous arteries. On the other hand, the high origin RA has the same origin as the former, but it continues in the forearm just as a normal RA, which is the only missing branch of BA in such cases. 

In 2017, M. Ozgur et al. [14] reported a case of high origin of RA from 3rd part of AA in a female cadaver. The course and branches of the artery were similar to the present case. It coursed along the medial aspect of the arm and continued laterally between the biceps and brachialis and more distally in the forearm, medial to brachioradialis. No branches were given in the arm. The AA continued as the BA, giving rise to UA and the common interosseous artery in the cubital fossa. The RA contributed to the formation of the superficial palmar arch in the hand.

The prevalence of high origin of RA from AA, as described in various literature, varied from 0.67% to 3.7%. The prevalence of high origin of RA from AA, as reported by various authors, is summarized in the table (Table 1) below.

**Table 1:** Comparison of prevalence of high origin of RA from AA in the present study to published literature * Angiographic study RA: radial artery; AA: axillary artery

Author(s)	Year of Study	Number of upper limbs studied	Prevalence of high origin of RA from AA (Number and Percentage)
Quain [15]	1844	429	16 (3.7%)
Adachi [16]	1928	410	9 (2.2%)
McCormack et al. [8]	1953	750	16 (2.13%)
Karlsson and Niechajev [17]	1982	164	2 (1.22%)
Uglietta and Kadir^*^ [6]	1989	100	1 (1%)
Rodriguez-Baeza et al. [18]	1995	150	1 (0.67%)
Rodriguez-Niedenführ et al. [19]	2001	384	12 (3.1%)
Nasr [20]	2012	100	1 (1%)
Haladaj et al. [21]	2018	120	2 (1.67%)
Present Study	2023	60	4 (6.70%)

In a study conducted by Haladaj et al. [21] in 120 upper limbs, the RA had a high origin in 11 cases, out of which only two were arising from the AA, i.e., 1.67% of all the dissected upper limbs. Both these were observed in male right limbs, and the brachioradial artery branched off the anterior aspect of the AA and ran anterior to the roots of the MN. Six of 11 brachioradial arteries observed anastomoses of brachioradial with 'normal' BA in the cubital fossa designated as cubital cross-overs or connections. They classified cubital cross-overs as dominant, balanced, and minimal depending on their diameter.

Previous studies reported anastomoses between brachioradial and BA at the cubital fossa in 19 - 50 % of upper limbs where a brachioradial artery was present [19]. In their study, M. Rodriguez-Niedenfuhr et al. [19] observed the brachioradial artery to be posterior to bicipital aponeurosis more often than anterior to it. Gonzalez-Compta [9]reported a piercing of bicipital aponeurosis, but we have observed no such cases. 

M. Rodriguez-Niedenfuhr et al. [19] also stated that in limbs with brachioradial artery, radial recurrent artery originated most commonly from the brachioradial, followed by the BA, and finally from the anastomosis between both vessels. However, when an anastomosis between a brachioradial and a BA is present, the radial recurrent arises more frequently from the anastomosis. 

In the present study, all the high-originating radial arteries were of the type "f" described by Haladaj [21]. There was no anastomosis between brachioradial and BA in the cubital fossa. The RA continued deep to the bicipital aponeurosis and the brachioradialis to the wrist and divided into superficial and deep branches, which completed the respective arterial arches of the palm by anastomosing with terminal branches of the UA. The radial recurrent artery was observed to arise from the BA proper in all four limbs with high originating RA from the AA. 

Three of four limbs with a high origin of RA from the AA were associated with other variations. In the case with bilateral high origin of RA from 3rd part, there was an associated common trunk for SSA and PCHA from a third part on both limbs. In one of the limbs, the LTA was arising from RA, which originated from the second part. 

Deviation from the regular embryological development of the vascular plexus of the limb bud results in variations and anomalies of the arterial supply of the upper limb [22]. The vascular plexus of the limb is supplied by four or five intersegmental branches of the dorsal aortae initially. The lateral branch of the seventh cervical intersegmental artery enlarges and gives rise to the axial artery of the upper limb, which later differentiates into the AA, BA, and interosseous arteries. The Axis artery connects the subclavian trunk with the capillary plexus of the arm buds. RA and UA are established later in the development [23-27]. Segmental arteries from C6, C7, and T1 levels and most longitudinal anastomosis linking the intersegmental arteries degenerate slowly. Anomalous arterial branching pattern results from the numerous alternatives existing during its formation [28] 

During development, the principal arteries, anastomose, and periarticular networks of capillaries emerge according to a temporal sequence; some initially functionally dominant paths regress subsequently. Differences in the mode and proximodistal branching level, aberrant vessels anastomosing with principal vessels, and vessels forming unexpected neural, myological, or osteo-ligamentous relationships lead to anomalous patterns [1,29]. Arrest at any stage of vessel development followed by regression, retention, or reappearance can lead to variation in arterial origin and branching patterns of major upper limb vessels [23,25]. 

Axilla being a crowded conduit for vasculatures and nerves passing between the trunk and upper extremities, extreme shoulder extension or depression during intrauterine development may cause compression and decreased space for normal development, resulting in unusual fusion and absorption of vascular plexus leading to variations and anomalies [30]. 

Early embryogenesis is also affected by genetic factors. Genetic factors, such as Unc5B, determine the identity and positioning of blood vessels in the early stages, whereas in the later part of embryogenesis, blood flow influences the remodeling of the blood vessels [31].

Gradual sprouting of arterial trunks takes place from a primitive axial artery. M. Rodriguez-Niedenfuhr et al. [19] proposed that upper limb arteries are formed by the union of superficial and deep pathways and presented the Superficial Brachial artery as a consistent embryonic vessel. As observed in the present study, the high origin of RA could be due to persistent Superficial Brachial artery. Newer theories explain that limb arteries develop by isolating main arterial trunks within the primitive capillary plexus. 

The limitation of the study was that a limited number of samples were taken in the present study, as per availability. Further studies on larger sample sizes, including retrospective angiographic studies, are recommended. Moreover, high originating RA from the BA was not considered as our study was confined to the branching patterns of AA.

## Conclusions

Anatomical variations of radial - brachial- axillary- subclavian arterial axis and arch of the aorta can lead to the failure of trans-radial coronary procedures and reconstructive upper limb surgeries. Ultrasonographic and contrast angiographic studies should be considered as a preoperative investigation for identifying such vascular anomalies to avoid accidental iatrogenic complications or failure of invasive procedures. Therefore, adequate anatomical information on the radial artery should help perform the trans-radial coronary procedure.
